# Association between estimated glucose disposal rate and cognitive impairment in elderly patients with type 2 diabetes mellitus: a cross-sectional study

**DOI:** 10.3389/fendo.2026.1690654

**Published:** 2026-02-18

**Authors:** Tong Chen, Hui-Na Qiu, Xin-Ping Zhang, Fan Wu, Yan-Lan Liu, Jing-Bo Li, Jing-Na Lin

**Affiliations:** 1 School of Medicine, Nankai University, Tianjin, China; 2 Department of Endocrinology, Tianjin Union Medical Center, The First Affiliated Hospital of Nankai University, Tianjin, China; 3 Department of Traditional Chinese Medicine, Peking University BinHai Hospital, Tianjin, China

**Keywords:** cognitive impairment, elderly patients, estimated glucose disposal rate, insulin resistance, type 2 diabetes mellitus

## Abstract

**Background:**

Insulin resistance (IR) is a fundamental pathophysiological characteristic of type 2 diabetes mellitus (T2DM) and is intricately related to neurodegeneration. This study sought to investigate the correlation between estimated glucose disposal rate (eGDR), an easily accessible and effective indicator of IR, and cognitive impairment (CI) in elderly patients with T2DM.

**Methods:**

This cross-sectional study included 871 elderly patients with T2DM. The eGDR was calculated from glycated hemoglobin (HbA1c), waist circumference and hypertension status to evaluate the extent of IR in patients. Cognitive function was assessed in all participants utilizing the Montreal Cognitive Assessment (MoCA). Linear and logistic regression analyses were performed to evaluate the association between eGDR and cognitive function. Restricted cubic spline (RCS) analysis and threshold effect analysis were conducted to elucidate the nonlinear relationship between eGDR and CI.

**Results:**

The eGDR levels were significantly lower in the CI group compared to the normal cognition group. Linear regression analysis indicated that eGDR was positively correlated with MoCA scores when expressed as continuous or categorical data after fully adjusting for covariates. Logistic regression analysis revealed that, after adjusting for all covariates, each unit increase in eGDR was associated with an 8% reduction in the risk of CI (OR: 0.92, 95% CI: 0.85-0.99, *P* < 0.05). Participants in the highest eGDR quartile exhibited a 38% lower risk of CI compared to those in the lowest eGDR quartile (OR: 0.62, 95% CI: 0.39-0.99, *P* < 0.05). RCS analysis and threshold effect analysis demonstrated a nonlinear relationship between eGDR and CI (*P* for non-linearity=0.001). When eGDR was<6.36 mg/kg/min, the risk of CI decreased with increasing eGDR levels (OR: 0.72, 95% CI: 0.61-0.86, *P* < 0.001). However, no significant association was observed when eGDR was≥6.36 mg/kg/min. Sensitivity analysis revealed a significant linear positive correlation between homeostatic model assessment (HOMA)2-IR and the risk of CI.

**Conclusion:**

In elderly patients with T2DM, eGDR is significantly associated with cognitive function, exhibiting a nonlinear relationship with the risk of CI. This finding provides novel insights for the prevention and management of CI.

## Introduction

With the acceleration of global population aging, the prevalence of type 2 diabetes mellitus (T2DM) has been steadily increasing, emerging as a significant public health concern impacting the health of the elderly population. Statistics indicate that approximately 828 million adults worldwide were diagnosed with diabetes in 2022, reflecting an increase of around 630 million since 1990. In China, approximately 148 million adults have diabetes, accounting for 18% of the global diabetes population (1). Epidemiological data from China reveal that the prevalence of diabetes among adults has reached 12.8%, escalating to 28.8% in individuals aged 60 and older (2). Diabetes markedly elevates the risk of mild cognitive impairment (MCI) and dementia (3, 4), and accelerates the progression of MCI to Alzheimer’s disease (AD) and dementia (5, 6). A community-based study conducted in China reported that the prevalence of dementia and MCI among individuals aged 60 years and older with diabetes was 10.2% and 18.5%, respectively (7). Cognitive impairment (CI) and dementia are increasingly recognized as complications and comorbidities associated with diabetes (8). Nevertheless, the precise diagnosis and management of CI in T2DM patients continue to pose significant challenges. Ding et al. underscored that the initial year following the diagnosis of MCI represents a critical window of opportunity for intervention, accentuating the significance of early identification and implementation of therapeutic measures (5). Exploring the relationship between T2DM-related factors and CI is of paramount importance for the development of effective prevention and intervention strategies.

Cognitive dysfunction associated with T2DM is influenced by multiple factors, and the specific underlying mechanisms remain poorly understood. Insulin resistance (IR) is a fundamental aspect of the pathophysiology of T2DM. Increasing evidence suggests that IR is not only associated with metabolic disorders but also plays a significant role in cognitive decline (9, 10). IR refers to the diminished sensitivity of insulin target tissues to insulin, with brain IR considered pivotal in diabetes-related cognitive dysfunction (11). Traditionally, the gold standard for evaluating IR is the hyperinsulinemic-euglycemic clamp. Although it is highly accurate, this technique is invasive, complex to perform and time-consuming, rendering it unsuitable for large-scale clinical studies and applications (12). In contrast, the homeostasis model assessment of IR (HOMA-IR), a commonly used alternative metric, is derived from fasting blood glucose and insulin levels. Despite its simplicity and convenience, HOMA-IR has inherent limitations, notably its susceptibility to fluctuations in fasting insulin levels, which reduces its efficacy in diabetic patients receiving insulin therapy (13). The estimated glucose disposal rate (eGDR) has garnered considerable attention in recent years as a non-invasive alternative indicator for assessing IR that does not require insulin measurement. eGDR is determined using readily accessible clinical markers, including waist circumference (WC), hypertension status and glycated hemoglobin (HbA1c). It was originally employed to evaluate insulin sensitivity in type 1 diabetes (T1DM) and demonstrates a high correlation with the results of the hyperinsulinemic-euglycemic clamp test (14). Studies have shown that eGDR can also function as an indirect indicator of IR in T2DM (15). Additionally, research has confirmed that eGDR possesses strong predictive value for cardiovascular disease (16) and is associated with diabetes-related complications (17–20) as well as cognitive dysfunction (21). Consequently, eGDR has potential applications not just in assessing IR but also in evaluating broader health outcomes. Nonetheless, existing studies examining the relationship between eGDR and cognitive function in T2DM patients remain insufficient, particularly within the elderly population.

This study aims to investigate the potential association between eGDR and CI in elderly T2DM patients, thereby addressing the existing research gap. Furthermore, it seeks to enhance the understanding of the connection between IR and CI in T2DM patients and to provide scientific evidence that may guide cognitive health management strategies for elderly T2DM patients.

## Materials and methods

### Study design and participants

The sample size was estimated using PASS V.15 software. According to previous studies, the prevalence of MCI among elderly patients with T2DM is 18.5% (7). Based on the simple asymptotic method, a two-tailed symmetric confidence interval was specified, with a confidence level of 0.95 and a confidence interval width of 0.1. Accounting for a 20% dropout rate, the final required sample size was estimated to be at least 290 participants. This cross-sectional study initially recruited 1,705 patients with T2DM who were hospitalized in the Department of Endocrinology at Tianjin Union Medical Center between July 2018 and June 2024. All participants met the 1999 WHO recommended diagnostic criteria for T2DM and were conscious and capable of completing the questionnaire. Exclusion criteria included: age<60 years; severe impairments in hearing, vision, reading, language or other reasons that could hinder the completion of cognitive assessments; missing data on key variables; anemia (Hb ≤ 90 g/L), severe hepatic or renal dysfunction; history of severe psychiatric disorders that could affect cognitive function (e.g., schizophrenia or major depressive disorder), malignant tumors, Parkinson’s disease, epilepsy, traumatic brain injury, or brain tumors.

**Figure 1:**
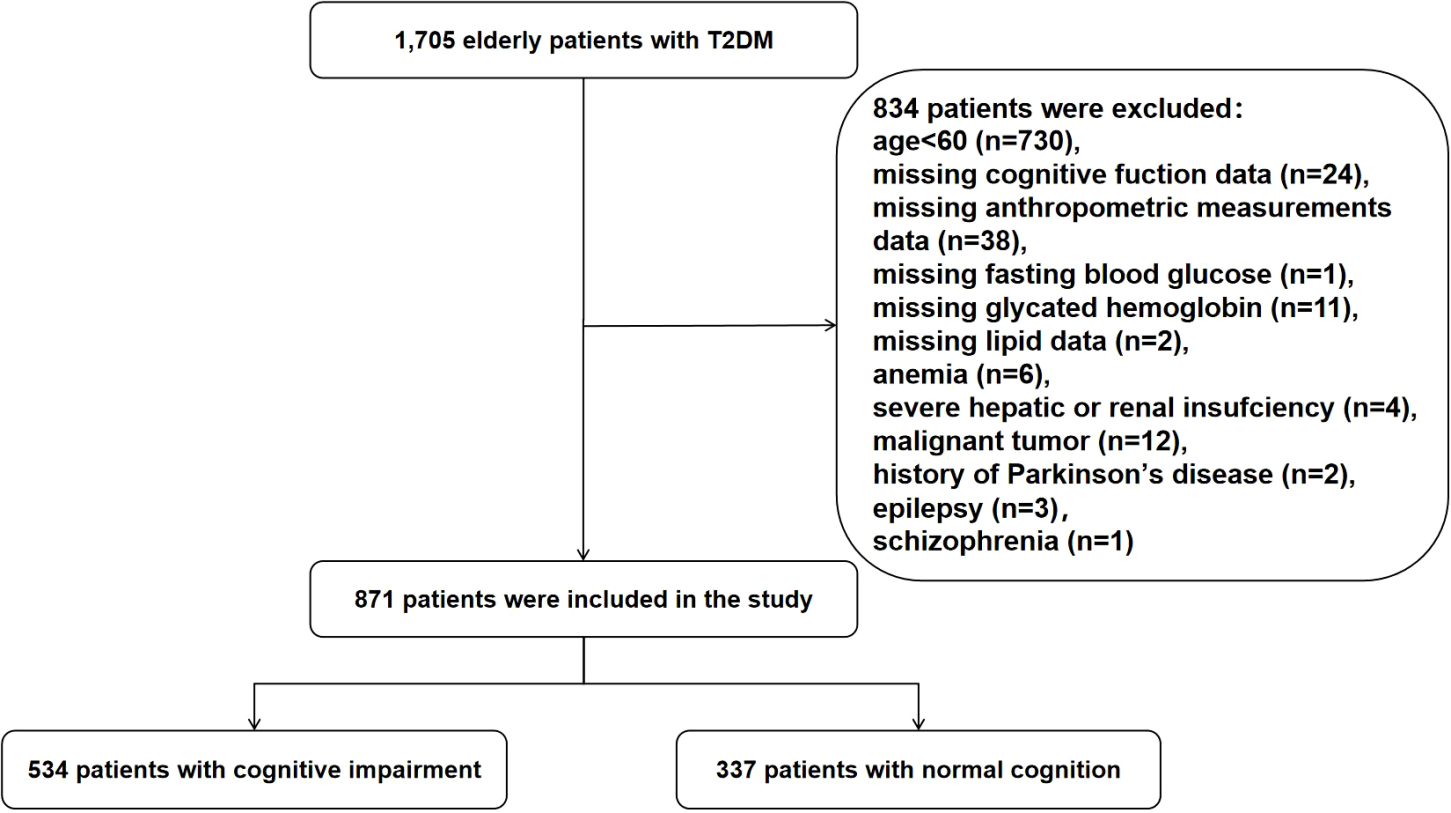
Flowchart of participant selection. T2DM, type 2 diabetes mellitus.

A total of 871 patients met the inclusion criteria and were included in the final analysis. The flowchart illustrating the selection process for study participants is provided in **Figure 1**. All participants provided written informed consent. The study was approved by the Medical Ethics Committee of Tianjin Union Medical Center and conducted in accordance with the Declaration of Helsinki.

### Data collection

Data were collected by trained clinical endocrinologists. The collected data encompassed demographic characteristics, diabetes-related information, anthropometric measurements and biochemical indicators.

Demographic characteristics included gender, ethnicity, age, marital status, education level, smoking status and alcohol consumption status. Diabetes-related variables included duration of T2DM, physical activity, nutritional management, antidiabetic medications and comorbidities including coronary artery disease (CAD), cerebrovascular disease (CVD) and hypertension. Physical activity was defined as engaging in at least 150 minutes of moderate-intensity aerobic exercise per week. Nutritional management referred to the regular intake of balanced calories and essential nutrients under the guidance of a diabetes manager.

Anthropometric measurements included height, weight, WC, body mass index (BMI) and visceral fat area (VFA). Height and weight were measured by health professionals using an automatic height and weight measurement device (DST-600, DONGHUAYUAN, China). WC was measured at the midpoint between the lower ribs and iliac crest utilizing a soft tape. BMI was calculated as weight (kg) divided by the square of height (m^2^). VFA was measured via direct segmental multi-frequency bioelectrical impedance analysis (DSM-BIA) (InBody720; Biospace Co, Ltd, Seoul, Korea).

Biochemical indicators included fasting plasma glucose (FPG), alanine aminotransferase (ALT), aspartate aminotransferase (AST), total cholesterol (TC), triglycerides (TG), high-density lipoprotein cholesterol (HDL-c) and low-density lipoprotein cholesterol (LDL-c). HbA1c was measured using the ARKRAY HA-8180 fully automated glycohemoglobin analyzer.

### eGDR assessment

eGDR was calculated using a formula incorporating three variables: HbA1c, WC and hypertension status (16). The specific formula used was as follows: eGDR = 21.158 - 0.09 * WC (cm) - 3.407 * Hypertension (yes=1/no=0) - 0.551 * HbA1c (%).

Hypertension was defined as systolic blood pressure (SBP) ≥140 mmHg or diastolic blood pressure (DBP) ≥90 mmHg, currently taking antihypertensive medications, or self-reported diagnosis of hypertension. A lower eGDR score indicated a higher degree of IR.

### Cognitive assessment

Cognitive function was assessed through face-to-face interviews conducted by trained researchers. The Chinese version of the Montreal Cognitive Assessment (MoCA-Beijing), which has been validated for the Chinese elderly population, was employed as a standardized tool for assessing cognitive function (22). The MoCA evaluates 11 items across 8 cognitive domains, including attention, executive function, delayed memory, language, visuospatial abilities, abstract thinking, calculation and orientation (23). The total score ranges from 0 to 30, with a score of less than 26 indicating CI (24). Participants were categorized into the CI group and the normal cognition group based on MoCA scores.

### Statistical methods

Continuous variables were expressed as mean ± standard deviation (SD) or median (25th-75th percentile), while categorical variables were presented as frequency and percentage [n (%)]. To compare differences between groups, Student’s t test (for normally distributed continuous variables), Mann-Whitney U test (for non-normally distributed continuous variables) or chi-square test (for categorical variables) were used. Linear regression analysis was performed to examine the correlation between eGDR and MoCA scores, while logistic regression analysis was applied to evaluate its relationship with CI. eGDR was analyzed both as a categorical variable and as a continuous variable. During the analysis, we adjusted for potential confounding variables and fitted three models. Model 1 was unadjusted. Model 2 was adjusted for gender, ethnicity, age, years of education, marital status, smoking status, alcohol consumption status, physical activity, nutritional management and duration of T2DM. Model 3 was further adjusted for CAD, CVD, ALT, HDL and BMI. For each model, regression coefficients (β) or odds ratios (OR) with corresponding 95% confidence intervals (95%CI) were calculated. Furthermore, we used multivariable restricted cubic spline (RCS) analysis to explore possible linear or nonlinear associations between eGDR and CI and establish a dose-response relationship model between the two variables. When nonlinearity was detected, piecewise regression model and likelihood ratio tests were employed to analyze the threshold effects, and a recursive algorithm was used to estimate the inflection point. To assess model stability, we conducted subgroup analyses and interaction analyses. These subgroups comprised gender, physical exercise, nutritional management, HbA1c, CVD and diabetes duration.

Additionally, we performed sensitivity analyses. An alternative IR indicator was used to further explore its association with cognitive function. To avoid interference from exogenous insulin due to some participants receiving insulin therapy, we used HOMA2-IR for a more accurate evaluation of IR. HOMA2-IR was calculated using the HOMA2 calculator based on FPG and fasting C-peptide (FCP) concentrations. The calculator can be accessed at https://homa-calculator.informer.com/2.2/. FPG and FCP levels were measured using radioimmunoassay. All statistical analyses were performed using R Version 4.3.2, and all *P* values were two-tailed. A *P*-value of <0.05 was considered statistically significant.

## Results

### Characteristics of participants

The study included a total of 871 patients aged 60 years and older with T2DM, of which 46.73% were male and 53.27% were female. The mean age of these patients was 66.89 ± 5.13 years. **Table 1** summarizes the demographic and clinical characteristics of elderly T2DM patients, among whom 534 participants (61.31%) had CI. CI was more common in female patients. Compared with participants with normal cognitive function, those with CI were older, had lower educational attainment, were less likely to engage in physical exercise and nutritional management, and showed greater rates of unmarried status, insulin use and CVD. In addition, patients in the CI group exhibited reduced levels of BMI, HDL, eGDR and ALT.

**Table 1:** Demographic information and clinical characteristics of the study population.

Variables	Total	Normal cognition	CI	*P*
n, %	871	337 (38.69%)	534 (61.31%)	
Gender				0.010
Female, n (%)	464 (53.27%)	161 (47.77%)	303 (56.74%)	
Male, n (%)	407 (46.73%)	176 (52.23%)	231 (43.26%)	
Han, n (%)	798 (91.62%)	311 (92.28%)	487 (91.20%)	0.573
Age, years	66.89 ± 5.13	66.20 ± 4.95	67.32 ± 5.20	0.002
Education				0.003
Below primary school, n (%)	11 (1.26%)	2 (0.59%)	9 (1.69%)	
Primary school, n (%)	54 (6.20%)	15 (4.45%)	39 (7.30%)	
Middle school, n (%)	378 (43.40%)	130 (38.58%)	248 (46.44%)	
High school or above, n (%)	428 (49.14%)	190 (56.38%)	238 (44.57%)	
Marital status				< 0.001
Single/divorced/widowed, n (%)	119 (13.66%)	29 (8.61%)	90 (16.85%)	
Married, n (%)	752 (86.34%)	308 (91.39%)	444 (83.15%)	
Smoking status				0.389
Never, n (%)	523 (60.05%)	211 (62.61%)	312 (58.43%)	
Former, n (%)	129 (14.81%)	44 (13.06%)	85 (15.92%)	
Current, n (%)	219 (25.14%)	82 (24.33%)	137 (25.66%)	
Alcohol consumption status				0.618
Never, n (%)	593 (68.08%)	229 (67.95%)	364 (68.16%)	
Former, n (%)	92 (10.56%)	32 (9.50%)	60 (11.24%)	
Current, n (%)	186 (21.35%)	76 (22.55%)	110 (20.60%)	
Physical exercise, n (%)	568 (65.21%)	240 (71.22%)	328 (61.42%)	0.003
Nutritional management, n (%)	580 (66.59%)	254 (75.37%)	326 (61.05%)	< 0.001
Duration of T2DM				0.433
<5 years, n (%)	294 (33.75%)	107 (31.75%)	187 (35.02%)	
5–10 years, n (%)	172 (19.75%)	73 (21.66%)	99 (18.54%)	
>10 years, n (%)	405 (46.50%)	157 (46.59%)	248 (46.44%)	
Metformin, n (%)	340 (39.04%)	132 (39.17%)	208 (38.95%)	0.949
Insulin, n (%)	340 (39.04%)	116 (34.42%)	224 (41.95%)	0.027
CAD, n (%)	372 (42.71%)	152 (45.1%)	220 (41.20%)	0.256
CVD, n (%)	329 (37.77%)	99 (29.38%)	230 (43.07%)	<0.001
Hypertension, n (%)	623 (71.53%)	234 (69.44%)	389 (72.85%)	0.277
FPG, mmol/L	7.93 ± 2.60	7.99 ± 2.63	7.89 ± 2.58	0.558
ALT, U/L	17.70 (12.70, 25.60)	18.90 (14.00, 26.80)	16.80 (12.00, 24.60)	0.001
AST, U/L	19.99 ± 9.80	20.15 ± 9.86	19.89 ± 9.77	0.705
TC, mmol/L	4.77 ± 1.15	4.79 ± 1.10	4.76 ± 1.19	0.737
TG, mmol/L	1.47 (1.08, 2.03)	1.48 (1.08, 2.11)	1.46 (1.07, 1.96)	0.304
HDL-c, mmol/L	1.18 ± 0.31	1.21 ± 0.31	1.16 ± 0.31	0.022
LDL-c, mmol/L	3.02 ± 0.90	3.01 ± 0.83	3.02 ± 0.94	0.949
HbA1c, %	8.61 ± 2.05	8.51 ± 1.95	8.68 ± 2.11	0.221
BMI, kg/m^2^	25.82 ± 3.47	26.20 ± 3.42	25.58 ± 3.48	0.010
VFA, cm^2^	103.62 ± 28.89	104.07 ± 29.08	103.33 ± 28.79	0.712
WC, cm	92.58 ± 9.66	91.83 ± 9.30	93.05 ± 9.86	0.069
MoCA scores	24.19 ± 3.04	27.18 ± 1.10	22.31 ± 2.26	<0.001
eGDR, mg/kg/min	5.64 ± 2.09	5.84 ± 2.00	5.52 ± 2.13	0.026

Continuous data were expressed as mean ± standard deviation or median.

(25th-75th percentile), while categorical data were presented as frequency and percentage [n (%)]. CI, cognitive impairment; CAD, coronary artery disease; CVD, cerebrovascular disease; FPG, fasting plasma glucose; ALT, alanine aminotransferase; AST, aspartate aminotransferase; TC, total cholesterol; TG, triglycerides; HDL-c, high-density lipoprotein cholesterol; LDL-c, low-density lipoprotein cholesterol; HbA1c, glycated hemoglobin; BMI, body mass index; VFA, visceral fat area; WC, waist circumference; MoCA, Montreal Cognitive Assessment; eGDR, estimated glucose disposal rate.

### Association of eGDR and cognition in elderly patients with T2DM

Multiple regression analyses were performed to examine the relationship between eGDR and cognitive function, with adjustment for covariates. In the linear regression analysis, in the unadjusted model (Model 1), eGDR was positively associated with MoCA scores (β: 0.16, 95% CI: 0.06-0.25). In the fully adjusted model (Model 3), the positive correlation between eGDR and MoCA scores remained statistically significant (β: 0.16, 95% CI: 0.06-0.26) (**Table 2**). When eGDR was categorized into quartiles, participants in the Q2 (β:0.58, 95% CI: 0.02-1.15), Q3 (β: 0.97, 95% CI: 0.40-1.54) and Q4 (β: 0.99, 95% CI: 0.42-1.55) groups exhibited higher MoCA scores compared with those in the Q1 group (**Table 2**). These associations persisted after adjusting for all covariates, suggesting that elevated eGDR levels may be associated with enhanced cognitive function.

**Table 2:** Relationship between eGDR and MoCA scores (linear regression analysis) and CI (logistic regression analysis).

eGDR	Linear regression analysis β (95% CI)			Logistic regression analysis OR (95% CI)		
	Model 1	Model 2	Model 3	Model 1	Model 2	Model 3
Continuous	0.16 (0.06, 0.25) **	0.08 (-0.02, 0.18)	0.16 (0.06, 0.26) **	0.93 (0.87, 0.99) *	0.98 (0.91, 1.05)	0.92 (0.85, 0.99) *
Q1	Ref	Ref	Ref	Ref	Ref	Ref
Q2	0.58 (0.02, 1.15) *	0.35 (-0.21, 0.90)	0.57 (0.02, 1.12) *	0.76 (0.51, 1.13)	0.92 (0.60, 1.40)	0.78 (0.50, 1.21)
Q3	0.97 (0.40, 1.54) **	0.86 (0.30, 1.42) **	1.24 (0.67, 1.82) **	0.51 (0.35, 0.76) **	0.55 (0.37, 0.84) **	0.40 (0.25, 0.63) **
Q4	0.99 (0.42, 1.55) **	0.49 (-0.08, 1.05)	0.96 (0.36, 1.55) **	0.64 (0.43, 0.95) *	0.88 (0.58, 1.35)	0.62(0.39, 0.99) *
*P* for trend	<0.001	0.030	<0.001	0.007	0.198	0.008

Model 1: unadjusted. Model 2: adjusted for gender, ethnicity, age, education, marital status, smoking status, alcohol consumption status, physical exercise, nutritional management and T2DM duration. Model 3: adjusted for gender, ethnicity, age, education, marital status, smoking status, alcohol consumption status, physical exercise, nutritional management, T2DM duration, CAD, CVD, ALT, HDL-c and BMI. eGDR, estimated glucose disposal rate; MoCA, Montreal Cognitive Assessment; CI, cognitive impairment; OR, odds ratio; 95% CI, 95% confidence intervals. *P < 0.05. **P < 0.01.

Logistic regression analysis was conducted to assess the relationship between eGDR and the risk of CI. The results showed a negative correlation between eGDR and CI in the unadjusted model (OR: 0.93, 95% CI: 0.87-0.99). After fully adjusting for covariates, this association remained statistically significant (OR: 0.92, 95% CI: 0.85-0.99) (**Table 2**). Further categorization of eGDR into quartiles showed that individuals in Q2, Q3 and Q4 groups had a lower risk of CI compared with the reference group (Q1 group), with ORs of 0.78 (95% CI: 0.50-1.21), 0.40 (95% CI: 0.25-0.63) and 0.62 (95% CI: 0.39-0.99), respectively (**Table 2**). These findings suggest that individuals with higher eGDR levels may have a lower risk of developing CI.

**Figure 2:**
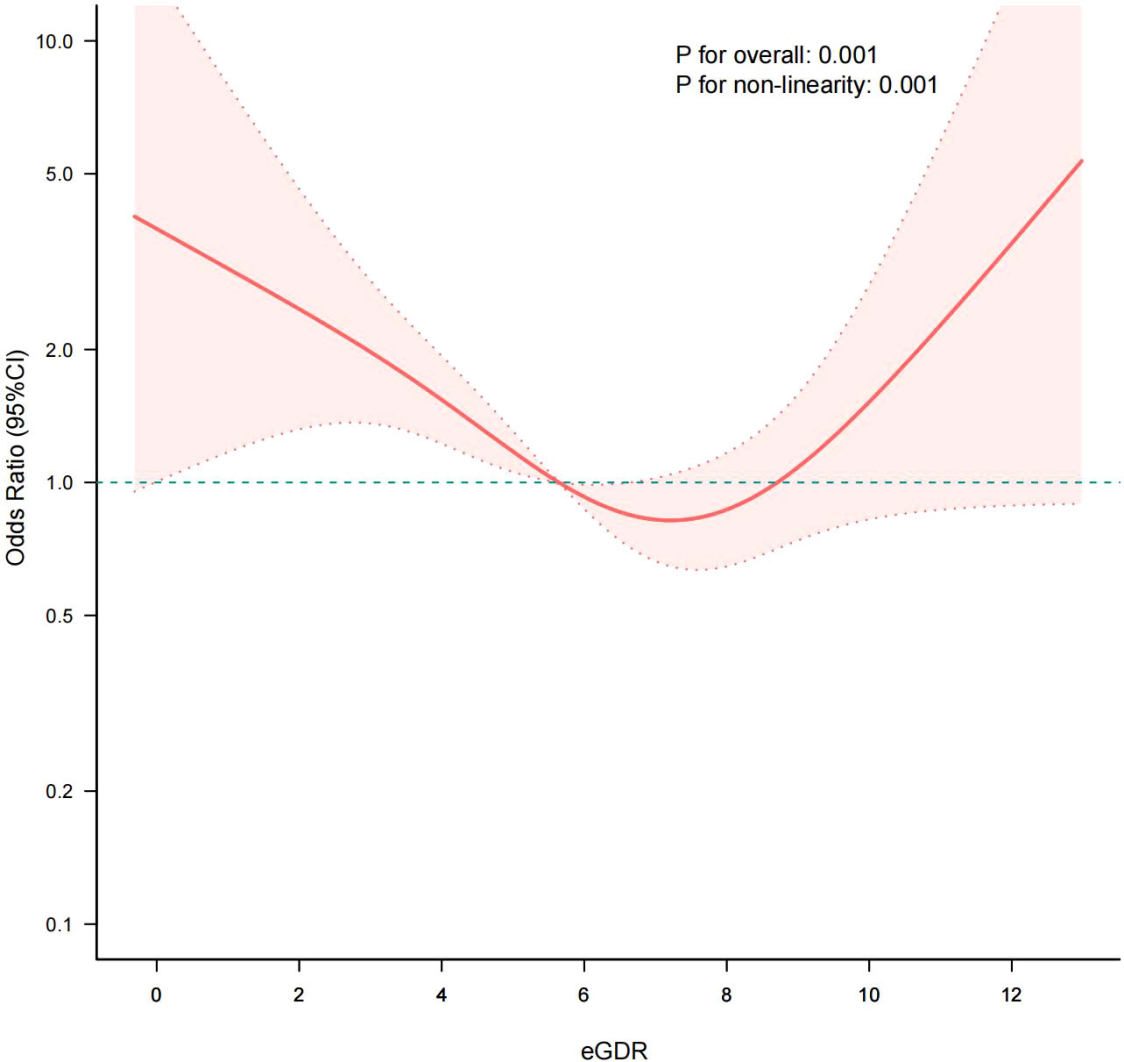
RCS analysis estimated the non-linear relationship between eGDR and CI. The solid lines and red areas represent the OR values and their corresponding 95% CIs, respectively. Adjusted for gender, ethnicity, age, education, marital status, smoking status, alcohol consumption status, physical exercise, nutritional management, T2DM duration, CAD, CVD, ALT, HDL-c and BMI. RCS, restricted cubic spline; eGDR, estimated glucose disposal rate; CI, cognitive impairment.

To further investigate the association between eGDR and CI, an RCS analysis was performed. The analysis revealed a significant nonlinear association after adjusting for covariates (*P* for nonlinear<0.001), with an inflection point identified at an eGDR of approximately 6.0 mg/kg/min (**Figure 2**). This suggests that lower eGDR levels, which indicate higher IR, are linked to an increased risk of CI up to a specific threshold, beyond which the relationship may reverse. Further threshold effect analysis using the likelihood ratio test revealed that the cut-off point for eGDR was 6.36 mg/kg/min. When eGDR<6.36 mg/kg/min, there was a negative correlation between eGDR and CI (OR: 0.72, 95% CI: 0.61-0.86, *P* < 0.001); however, when eGDR was≥6.36 mg/kg/min, the relationship between eGDR and CI was not statistically significant (OR: 0.72, 95% CI: 0.61-0.86, *P* < 0.001) (**Table 3**).

**Table 3:** Threshold effect analysis of eGDR and CI.

Outcome	OR (95%CI)	*P*
Inflection point	6.36	
<6.36	0.72 (0.61, 0.86)	<0.001
≥6.36	1.18 (0.94, 1.49)	0.159
*P* for likelihood test		0.001

Adjusted for gender, ethnicity, age, education, marital status, smoking status, alcohol consumption status, physical exercise, nutritional management, T2DM duration, CAD, CVD, ALT, HDL-c and BMI. eGDR, estimated glucose disposal rate; CI, cognitive impairment; OR, odds ratio; 95% CI, 95% confidence intervals.

### Subgroup analyses

**Figure 3:**
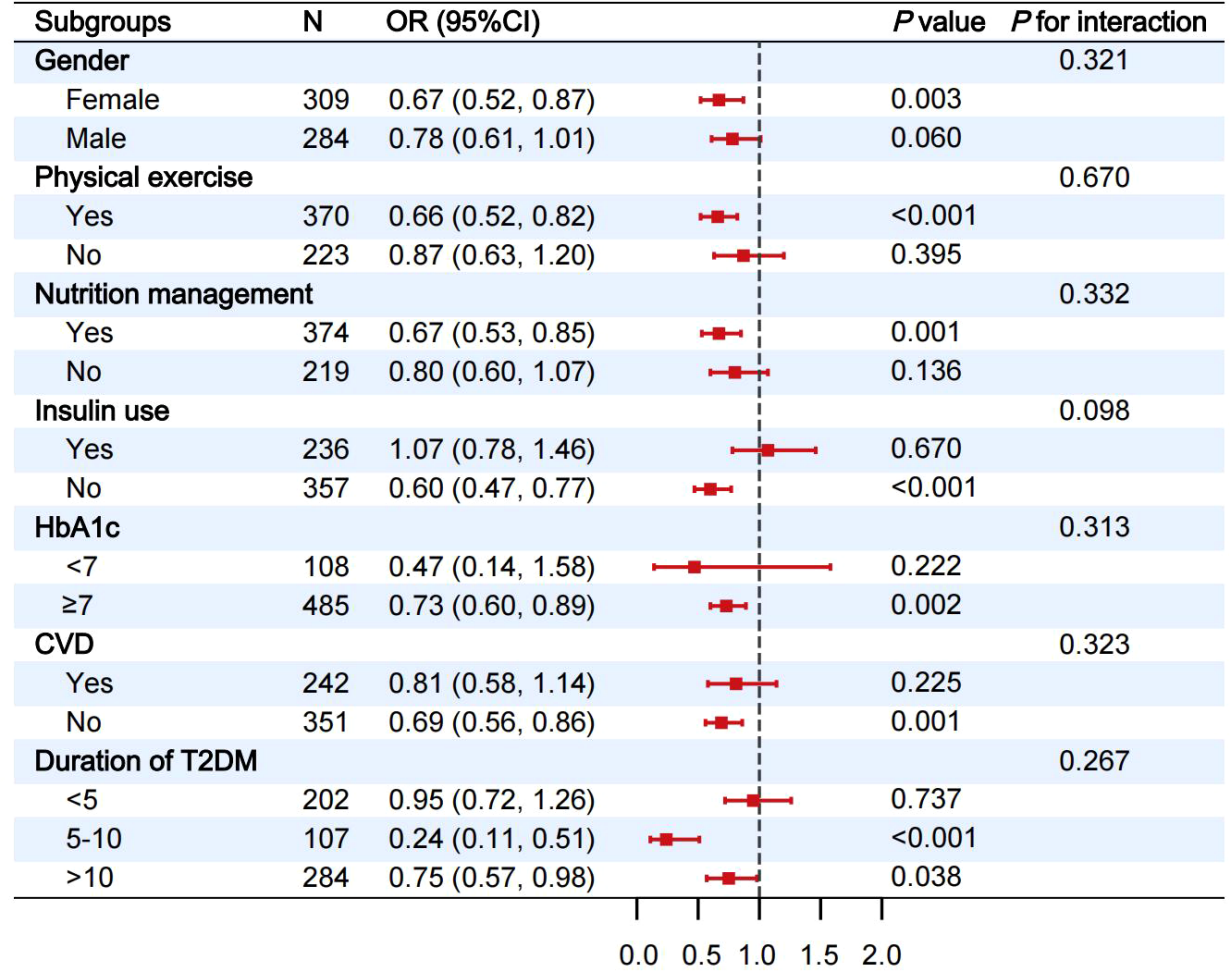
Stratified analysis of the relationship between eGDR and CI when eGDR was<6.36 mg/kg/min. Each subgroup analysis was adjusted for gender, ethnicity, age, education, marital status, smoking status, alcohol consumption status, physical exercise, nutritional management, T2DM duration, CAD, CVD, ALT, HDL-c and BMI, except for stratification variables. eGDR, estimated glucose disposal rate; CI, cognitive impairment; OR, odds ratio; 95% CI, 95% confidence intervals; HbA1c, glycated hemoglobin; CVD, cerebrovascular disease; T2DM, type 2 diabetes mellitus.

Subgroup and interaction analyses were further conducted to evaluate the stability of the observed associations. Given the nonlinear association between eGDR and CI, participants were stratified into two groups based on the identified threshold (6.36 mg/kg/min). The association between eGDR and CI was then assessed across subgroups defined by gender, physical exercise, nutritional management, insulin use, HbA1c, CVD and duration of T2DM (**Figure 3**). Among patients with eGDR<6.36 mg/kg/min, the inverse association between eGDR and the risk of CI remained statistically significant in several subgroups, including females, those engaged in physical exercise, those with nutritional management, those not receiving insulin therapy, individuals with HbA1c≥7%, those without CVD and those with a T2DM duration≥5 years. Interaction terms for these subgroups were not statistically significant (*P* for interaction>0.05), suggesting consistency across strata (**Figure 3**).

**Figure 4:**
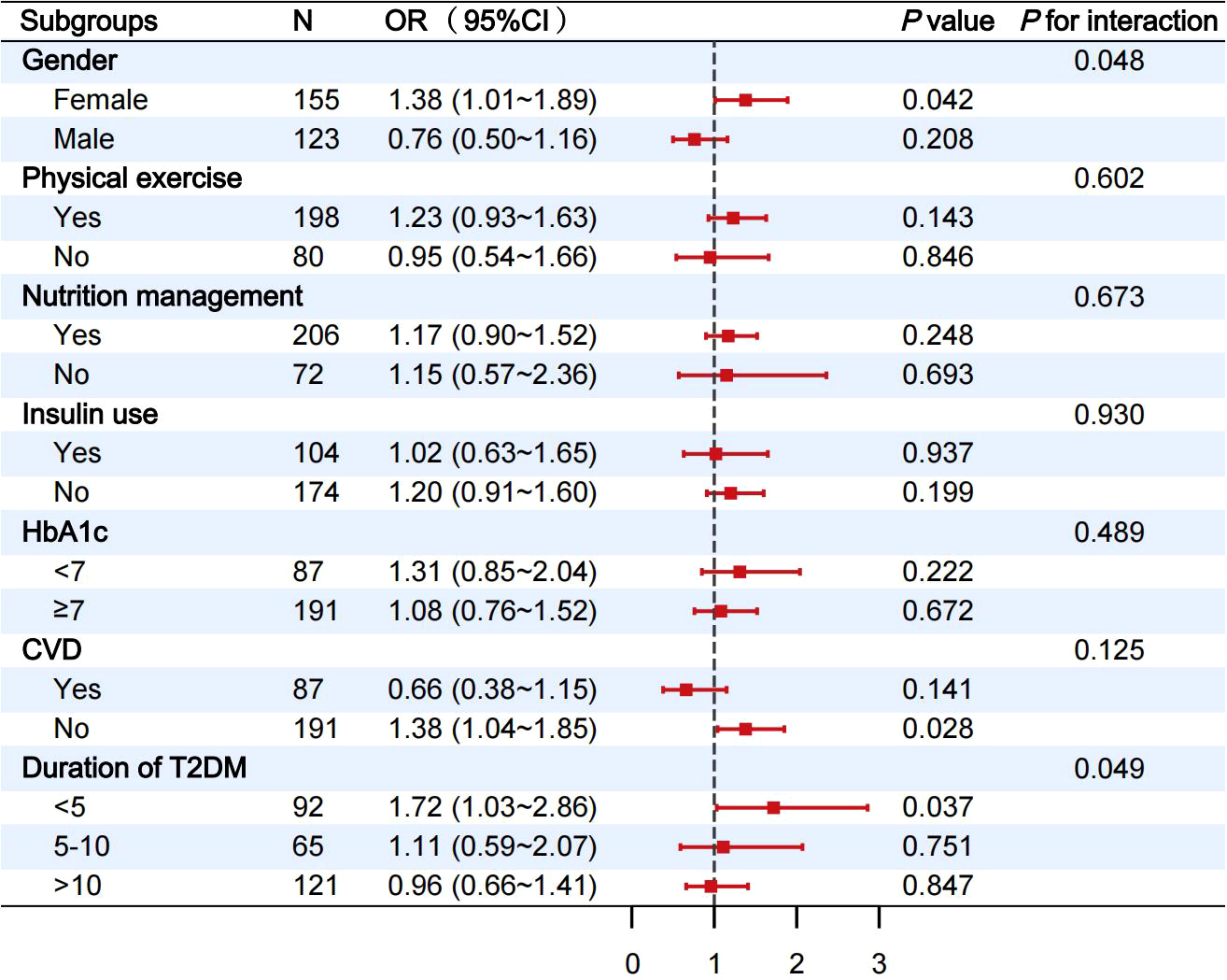
Stratified analysis of the relationship between eGDR and CI when eGDR was≥6.36 mg/kg/min. Each subgroup analysis was adjusted for gender, ethnicity, age, education, marital status, smoking status, alcohol consumption status, physical exercise, nutritional management, T2DM duration, CAD, CVD, ALT, HDL-c and BMI, except for stratification variables. eGDR, estimated glucose disposal rate; CI, cognitive impairment; OR, odds ratio; 95% CI, 95% confidence intervals; HbA1c, glycated hemoglobin; CVD, cerebrovascular disease; T2DM, type 2 diabetes mellitus.

Among participants with eGDR≥6.36 mg/kg/min, the association between eGDR and CI was consistent across most subgroups, including physical exercise, nutritional management, insulin use, HbA1c and CVD (all *P* for interaction>0.05) (**Figure 4**). However, interactions were observed for gender and DM duration categories (*P* for interaction < 0.05) (**Figure 4**).

### Sensitivity analyses

**Figure 5:**
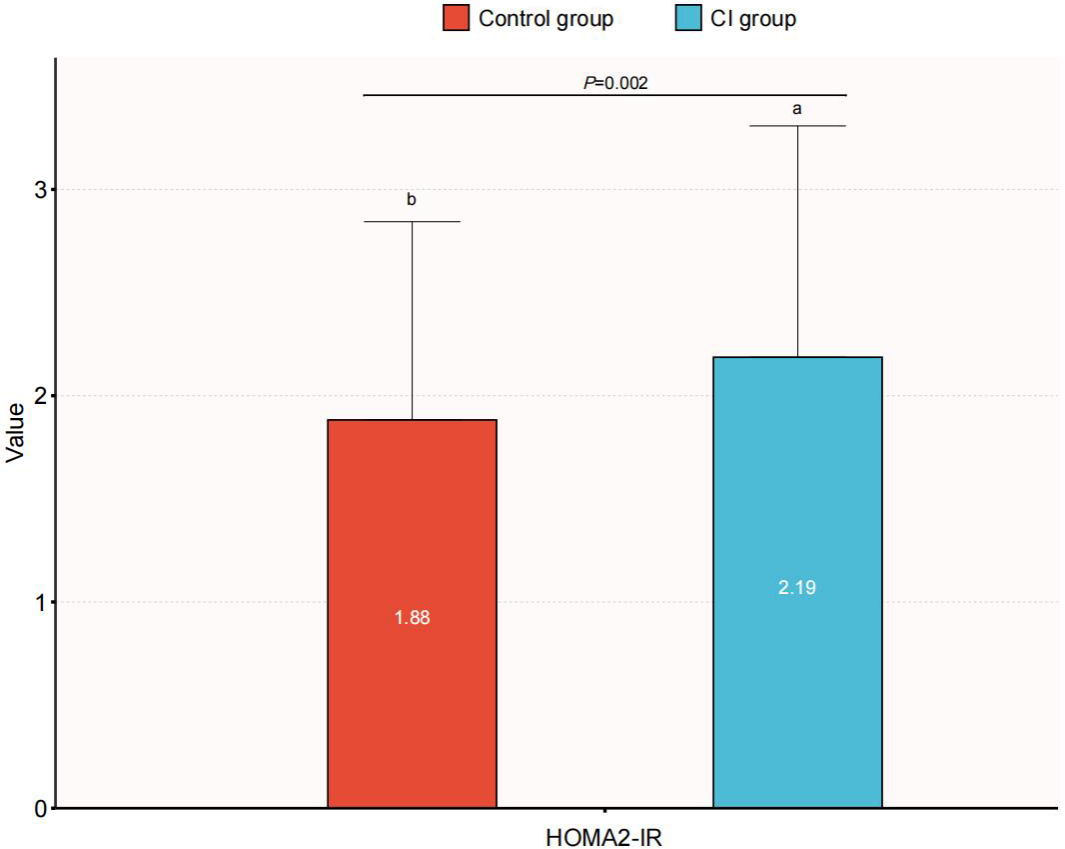
Comparison of HOMA2-IR between the CI group and the normal cognition group. CI, cognitive impairment.

**Figure 6:**
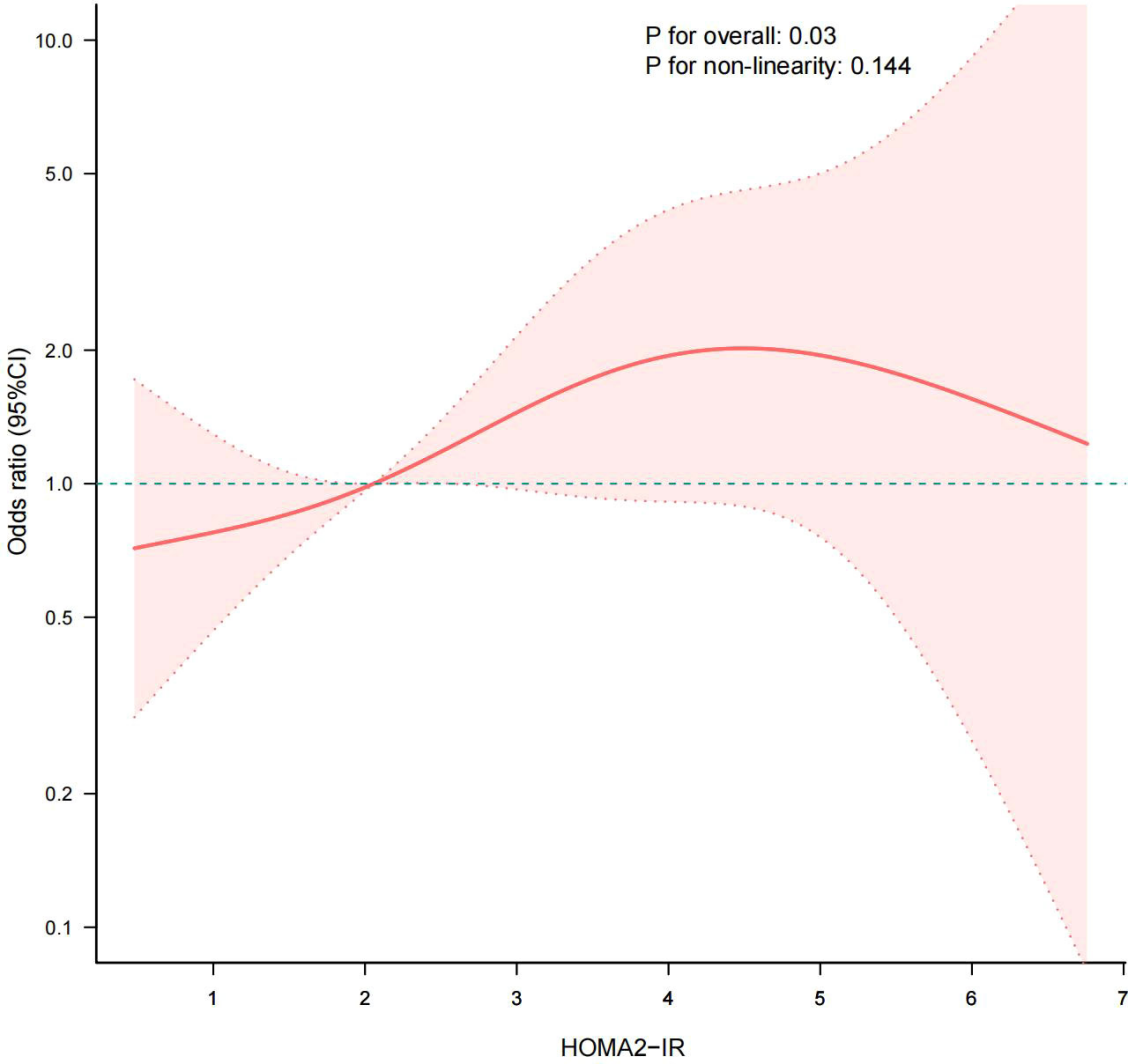
RCS analysis estimated the relationship between HOMA2-IR and CI. Adjusted for gender, ethnicity, age, education, marital status, smoking status, alcohol consumption status, physical exercise, nutritional management, T2DM duration, CAD, CVD, ALT, HDL-c and BMI. The solid lines and red areas represent the OR values and their corresponding 95% CIs, respectively. RCS, restricted cubic spline; CI, cognitive impairment.

We additionally conducted sensitivity analyses. Given that eGDR is a reliable surrogate marker for IR, we assessed another alternative IR indicator, HOMA2-IR, to reveal the association between IR and CI. After excluding participants with missing data on FPG and FCP, 457 elderly patients with T2DM were eligible for analysis, including 195 patients with normal cognition and 262 patients with CI. HOMA2-IR levels were significantly higher in the CI group compared to the normal cognition group, with a statistically significant difference between groups (*P* = 0.002) (**Figure 5**). **Table 4** shows the results of the multivariate logistic regression analysis of HOMA2-IR and CI. The results showed that in Model 1, HOMA2-IR was positively associated with the risk of CI (OR: 1.33, 95% CI: 1.10-1.60, *P* = 0.003). Although the association was not significant in Model 2, a significant association between HOMA2-IR and the prevalence of CI was still observed in the fully adjusted model (Model 3) (OR: 1.35, 95% CI: 1.08-1.70, *P* = 0.010). Further analysis by HOMA2-IR quartiles revealed that, using the lowest quartile (Q1) as the reference group, a significant association was observed in the highest quartile (Q4) (OR: 2.33, 95% CI: 1.18-4.60, *P* = 0.015), with a significant trend across quartiles (*P* for trend<0.05). Results of RCS analysis indicated a linear association between HOMA2-IR and the risk of CI (*P* for nonlinear=0.144) (**Figure 6**). These findings suggest that the risk of CI increased linearly with rising HOMA2-IR levels.

**Table 4:** Association between HOMA2-IR and CI in elderly patients with T2DM.

	Model 1		Model 2		Model 3	
HOMA2-IR	OR (95% CI)	*P* value	OR (95% CI)	*P* value	OR (95% CI)	*P* value
Continuous	1.33 (1.10, 1.60)	0.003	1.20 (0.98, 1.48)	0.077	1.35 (1.08, 1.70)	0.010
Q1	Ref		Ref		Ref	
Q2	0.97 (0.57, 1.62)	0.895	0.99 (0.56, 1.77)	0.982	0.99 (0.54, 1.81)	0.968
Q3	1.56 (0.92, 2.65)	0.097	1.64 (0.91, 2.96)	0.099	1.82 (0.97, 3.43)	0.064
Q4	2.07 (1.21, 3.55)	0.008	1.72 (0.94, 3.16)	0.079	2.33 (1.18, 4.60)	0.015
*P* for trend		0.002		0.027		0.004

Model 1: unadjusted. Model 2: adjusted for gender, ethnicity, age, education, marital status, smoking status, alcohol consumption status, physical exercise, nutritional management, T2DM duration. Model 3: adjusted for gender, ethnicity, age, education, marital status, smoking status, alcohol consumption status, physical exercise, nutritional management, T2DM duration, CAD, CVD, ALT, HDL-c and BMI. CI, cognitive impairment; T2DM, type 2 diabetes mellitus; OR, odds ratio; 95% CI, 95% confidence intervals.

## Discussion

In this cross-sectional study of elderly Chinese patients with T2DM, we investigated the association between eGDR and CI, and additionally examined the relationship between the traditional IR indicator, HOMA2-IR, and CI as part of a sensitivity analysis. A significant association between eGDR and CI was observed both before and after adjusting for covariates. RCS analysis demonstrated a nonlinear relationship, where decreasing eGDR paralleled an increasing risk of CI. A threshold effect analysis employing the maximum likelihood method identified an inflection point at an eGDR of 6.36 mg/kg/min after adjusting for gender, ethnicity, age, education, marital status, smoking status, alcohol consumption status, physical exercise, nutritional management, duration of T2DM, CAD, CVD, ALT, HDL-c and BMI. Subgroup analysis showed that in patients with eGDR<6.36 mg/kg/min, the association between eGDR and CI was more evident in women, those engaging in physical exercise, those with nutrition management, those not using insulin therapy, those with HbA1c≥7%, those without CVD, T2DM duration≥5 years; however, none of these subgroups exhibited a statistically significant interaction with eGDR (*P* for interaction>0.05). Additionally, a linear positive correlation between HOMA2-IR and CI was observed.

IR is a core pathophysiological mechanism of T2DM and has been extensively acknowledged for its role in metabolic disorders. It is also implicated as a substantial factor in T2DM-related cognitive dysfunction (25, 26). IR has been observed to correlate with altered cerebral blood flow in individuals with or without T2DM (27, 28). A recent comparative clinical trial revealed that both IR and hyperglycemia compromised cerebral hemodynamics and oxygenation in T2DM patients, potentially bearing implications for cognitive function and overall brain health (29). In this study, patients with CI exhibited significantly lower eGDR levels compared to those with normal cognitive function, and eGDR was significantly associated with cognitive function, further highlighting the pivotal role of IR in CI among elderly individuals with T2DM. Consistent with the findings of our study, Li et al. conducted a cross-sectional study analysis based on the National Health and Nutrition Examination Survey (NHANES) database, reporting a nonlinear relationship between eGDR and cognitive function in the elderly population (≥60 years old), with a positive association observed when eGDR was below 5.88 mg/kg/min, and a negative association beyond that threshold (21). Longitudinal data from the China Health and Retirement Longitudinal Study (CHARLS) further support the utility of eGDR as a promising biomarker for cognitive risk stratification in non-diabetic middle-aged and elderly populations (30). The triglyceride glucose (TyG) index, another surrogate marker for IR, derived from fasting TG and blood glucose, has demonstrated good sensitivity and specificity compared to the gold standard for IR assessment (31–33). A retrospective study by Teng et al. involving 308 elderly patients with T2DM found the TyG index to be an independent risk factor for both CI and cerebral small vessel disease (34). Another cross-sectional study based on a community population also reported that an elevated TyG index was significantly associated with higher CI risk (OR: 1.64, 95% CI: 1.02-2.63, *P* = 0.042) (35). In a comprehensive meta-analysis, Sandeep et al. found a significant association between TyG index and both CI (OR: 2.31, 95% CI: 1.38-3.86) and dementia (OR:1.14, 95% CI: 1.12-1.16) (36). Among individuals with normal cognitive function, elevated HOMA-IR has been associated with reduced cerebral glucose metabolism – a hallmark of AD (37). In this study, our sensitivity analysis further indicated that increased HOMA2-IR levels were significantly associated with higher CI risk, consistent with findings reported by Zhao et al. (38). The linear relationship between HOMA2-IR and CI differs from the nonlinear relationship of eGDR, indicating that eGDR may exhibit more sensitivity to changes in cognitive function within certain specific ranges. Collectively, these studies provide compelling evidence for the involvement of IR in the pathogenesis of CI. However, some reports, particularly those focusing on non-diabetic populations, have failed to demonstrate a significant association between IR (as assessed by HOMA-IR) and cognitive function (39, 40), indicating the need for further research to elucidate the heterogeneity in these findings.

Several mechanisms have been proposed to explain the association between IR and cognitive decline in patients with T2DM. First, IR may be involved in the pathophysiology of CI by disrupting glucose metabolism in the brain. Dysregulation of the insulin signaling pathway is associated with impaired glucose metabolism and impaired ATP production, which may be linked to neuronal apoptosis and dysfunction, thereby potentially playing a role in CI (11, 41). Second, IR may be related to vascular CI through its association with cerebral vascular dysfunction and structural changes. Hyperglycemia and IR often coexist with impaired vasodilation, vascular endothelium damage, accumulation of advanced glycation end products (AGEs) (42) and proinflammatory mediators, thereby potentially elevating the susceptibility to cerebral arteriosclerosis and vascular dementia. Third, IR may also be intertwined with amyloid-beta protein (Aβ) deposition and tau protein hyperphosphorylation, hallmark pathologies of AD, thereby potentially being associated with AD-related CI (38, 43). Fourth, IR may be associated with compromised neuronal and synaptic function through chronic inflammation and oxidative stress. Elevated levels of inflammatory cytokines, such as tumor necrosis factor-alpha (TNF-α) and interleukin-6 (IL-6), can activate microglia and elicit inflammatory responses, fostering blood-brain barrier disruption and neuronal damage (44–46). Our findings reveal that both eGDR and HOMA2-IR, as surrogate markers of IR, are significantly associated with CI.

This study showed a significant nonlinear correlation between eGDR and CI, with a threshold effect analysis revealing that 6.36 mg/kg/min was the turning point. Specifically, when eGDR was<6.36 mg/kg/min, a negative association with CI was observed; conversely, when eGDR was≥6.36 mg/kg/min, the association was not statistically significant. Identifying this specific threshold of 6.36 mg/kg/min may have potential utility in routine practice. Given that eGDR can be easily calculated from routinely collected clinical parameters, it may serve as a practical screening indicator to identify elderly patients with T2DM who are at higher risk of CI and who may benefit from early cognitive assessment and targeted interventions. Subgroup analyses revealed that the association between eGDR and CI risk varied across different patient subgroups with eGDR<6.36 mg/kg/min. In detail, the association was more pronounced in women, individuals engaging in physical exercise, those with nutritional management, those not using insulin therapy, those with HbA1c≥7%, those without CVD and those with a duration of T2DM ≥5 years, although no potential interactions were observed. Higher eGDR levels were associated with a reduced risk of CI in women, whereas this association was not significant in men. Consistent with our findings, Luo et al. conducted a similar sex-specific association using data from the CHARLS (47). This gender difference may be due to the combined influence of physiological, metabolic and social factors. The neuroprotective effects of estrogen weaken after menopause, potentially making women more vulnerable to cognitive decline observed in the context of IR (48, 49). The relationship between eGDR and CI was more significant in patients who did physical exercise and engaged in nutrition management, suggesting that a healthy lifestyle may weaken the correlation between IR and cognitive function to some extent. Exercise has been shown to improve insulin sensitivity, reduce IR, enhance cerebral blood flow and exert neuroprotective effects (50–52). Likewise, reasonable nutritional management helps stabilize blood glucose levels and improves metabolism, whereas unhealthy dietary patterns (e.g., high-fat or high-sugar intake) may be independently related to cognitive decline through neurotoxic or vascular damage (53–55), thereby modifying the primary correlation between eGDR and cognition. In patients not treated with insulin, the association between eGDR and CI was markedly more pronounced. Evidence indicates that patients with T2DM receiving intranasal insulin therapy experience increased cerebral blood flow, improved IR and beneficial effects on cognition (56). We consider that exogenous insulin therapy may partially compensate for the detrimental effects of endogenous IR or insufficient sensitivity on the brain. Moreover, patients receiving insulin therapy may represent a more intricate, longer-duration or more comorbid patient population, where IR is merely one of several pathogenic factors, thereby diminishing its independent effect. In addition, insulin may exert neuroprotective effects by acting on the insulin signaling pathway in the brain (57), potentially mitigating some damage induced by low eGDR. In patients with poor glycemic control (HbA1c≥7%), the association between eGDR and CI was more significant. This may be driven by the fact that in a persistently hyperglycemic environment, the adverse association of IR with cerebral microvascular damage (58) and neuroinflammation (59) may be more pronounced, becoming a more important factor related to CI. In elderly diabetic patients with relatively healthy cardiovascular systems, IR may be a relatively independent and more important pathophysiological mechanism contributing to cognitive decline. In patients with existing CVD, CVD is an important risk factor for CI (60) and may increasingly overshadow the impact of IR as an isolated component. The association between eGDR and CI was more evident in patients with a longer duration of T2DM (≥5 years), suggesting that the role of IR in the brain may be a chronic and cumulative process. Prolonged exposure to IR may be accompanied by sustained inflammation, oxidative stress, endothelial dysfunction and other deleterious effects that are tied to cognitive function (61). These findings underscore the importance of individualized interventions based on patient characteristics to mitigate the cognitive risks associated with IR in elderly T2DM populations.

This study possesses several noteworthy strengths. To the best of our knowledge, this is the first investigation to explore the association between eGDR and CI in elderly patients with T2DM. Furthermore, we employed another widely accepted surrogate marker of IR, HOMA2-IR, to validate the relationship between IR and CI. However, several limitations must be acknowledged. First, this study adopts a cross-sectional design. It is imperative to acknowledge that while we observed a significant correlation between eGDR and CI, the inherent limitations of this study preclude us from establishing a causal relationship or determining the direction of causality. Future longitudinal studies are warranted to further elucidate the temporal relationship between eGDR and cognitive decline. Second, although we adjusted for multiple covariates to the greatest extent possible, residual confounding factors may potentially affect the relationship between eGDR and cognitive function. Future research should consider incorporating additional potential confounding variables. Third, this study utilized a single cognitive assessment tool. While the MoCA is an effective screening tool, it does not provide detailed domain-specific cognitive profiles. Future studies should consider incorporating multiple neuropsychological tests to enhance the comprehensiveness and reliability of cognitive evaluation. Fourth, as this study was a single-center study, the generalizability and representativeness of the findings may be limited. Fifth, detailed data on hypoglycemic episodes and long-term glycemic variability were not available in this cross-sectional study, which are known factors influencing cognitive function (62, 63); therefore, their potential effects on cognitive function could not be evaluated in the study.

## Conclusions

In conclusion, our study demonstrates a nonlinear negative correlation between eGDR and CI in elderly T2DM patients. As a practical surrogate for IR, eGDR shows potential as a clinical tool for the early identification of individuals at risk for CI. Prospective studies are needed to further validate the predictive and interventional value of eGDR in the context of diabetes-related cognitive decline.

## Data Availability

The raw data supporting the conclusions of this article will be made available by the authors, without undue reservation.
